# Use of Nebulized Corticosteroids Beyond Asthma and Chronic Obstructive Pulmonary Disease (COPD): A Scoping Review of Off-Label Respiratory and Perioperative Applications

**DOI:** 10.7759/cureus.113068

**Published:** 2026-07-21

**Authors:** Meenu Singh, Gowrishankar NC, Rashmi R Das, Ashwin Jadhav, Meena Lopez, Jaideep Gogtay

**Affiliations:** 1 Pediatrics, All India Institute of Medical Sciences, Chandigarh, IND; 2 Pediatrics, Mehta Multispeciality Hospital, Chennai, IND; 3 Pediatrics, All India Institute of Medical Sciences, Bhubaneswar, IND; 4 Medical Affairs, Cipla Ltd., Mumbai, IND

**Keywords:** allergy, corticosteroid, infection, inflammation, nebulization, nebulizer, steroid

## Abstract

Current literature suggests the potential benefits of nebulized corticosteroids (NCs), particularly budesonide and fluticasone, in the treatment of various respiratory conditions beyond their conventional application. This review aimed to comprehensively summarize the available evidence regarding the use of NCs for medical conditions beyond their conventional role in asthma and chronic obstructive pulmonary disease (COPD) management. This scoping review included all studies evaluating the use of NCs in children and adults for indications other than asthma and COPD. The search was limited to human studies published in English. Data were collected from the PubMed and Cochrane databases through December 2025. A narrative approach was employed to synthesize the extracted data, which are summarized and presented in tables and graphs. As this was a scoping review, formal risk of bias assessment and certainty of evidence evaluation were not performed. A predefined methodological approach was followed; however, no formal protocol registration was undertaken. A total of 302 articles were identified from various databases, of which 36 met the inclusion criteria. The included studies enrolled a total of 3,415 patients (range: 20-525) and primarily comprised randomized controlled trials, including double-blind placebo-controlled studies, prospective randomized studies, multicenter trials, pilot studies, and comparative interventional studies. Several indications beyond the conventional use of NCs in asthma/COPD were identified, including bronchiolitis, transient tachypnea of the newborn, respiratory complications after endotracheal intubation, croup, meconium aspiration syndrome, chronic rhinosinusitis, and viral wheezing. Overall, the available literature suggests that NCs may have therapeutic applications beyond asthma and COPD; however, the available evidence was heterogeneous with respect to indications, patient populations, study designs, and outcome measures. Further research is needed to clarify the effectiveness and safety of NCs, enabling evidence-based decision-making and improving patient-related outcomes.

## Introduction and background

Corticosteroids play a crucial role in the treatment of allergic and inflammatory conditions and in controlling unwanted or inappropriate immune system responses [1]. Corticosteroids exert their anti-inflammatory effects by diffusing into cells and binding to intracellular glucocorticoid receptors, causing a conformational change and modulating gene transcription, leading to reduced production of proinflammatory mediators and overall suppression of inflammation [2].

Corticosteroids are commonly used in the fields of allergy and pulmonology to reduce airway hyperresponsiveness and airway inflammation, promote epithelial repair, and support the regeneration of airway cilia [1,3]. Their anti-inflammatory properties have also led to increasing investigation and use in other clinical settings, including perioperative airway management and selected upper airway conditions [4]. Their anti-inflammatory effects are mediated through multiple complementary mechanisms, including suppression of the infiltration of inflammatory cells, such as Langerhans cells, mast cells, eosinophils, and T cells; inhibition of cytokine-mediated signaling and delayed-type hypersensitivity responses; and reduction of mediator release from the arachidonic acid pathway. Collectively, these actions lead to decreased mucosal edema, reduced vascular permeability, and improved airway patency, thereby preventing airway obstruction. In addition, corticosteroids lower circulating eosinophil counts, inhibit eosinophil migration, and suppress eosinophil-specific cytokines while enhancing epithelial Na+ channel activity, which contributes to improved airway surface fluid regulation and respiratory function [2,4-6].

Corticosteroids can be administered via various routes, including oral, injectable (intramuscular, intradermal, intra-articular, and intralesional), inhalation, topical, rectal, and parenteral routes, primarily depending on the condition being treated [1]. Corticosteroids can also be delivered using nebulizers [7]. Nebulizers play an important role in administering a therapeutic dose of the drug within 15 minutes, ensuring effective delivery and distribution throughout the lungs in patients of all age groups [8]. In addition, nebulizers are easy to use and require minimal cognitive skills [9]. Nebulized corticosteroids (NCs) provide an alternative delivery route that may be particularly useful in patients who are unable to use handheld inhaler devices effectively or who require targeted airway drug delivery [10]. Nebulization allows passive inhalation of aerosolized medication, enabling deposition throughout the respiratory tract without the need for coordinated inhalation effort [11]. This approach may improve treatment accessibility in pediatric and older adult patients, as well as in those with acute or severe respiratory conditions. NCs include budesonide, fluticasone propionate, flunisolide, and beclomethasone dipropionate [12]. These drugs are recognized for their strong affinity for the glucocorticoid receptor, effective topical anti-inflammatory properties, and minimal likelihood of causing systemic effects [12].

Currently, substantial evidence supports the use of NCs to reduce morbidity and improve the overall health of patients with asthma and chronic obstructive pulmonary disease (COPD), which represent the conventional and most widely approved clinical indications for NCs [12-15]. However, because of their efficacy in reducing airway inflammation and obstruction, the use of NCs beyond their conventional role in asthma and COPD management has also been explored. Other conditions for which NCs have been prescribed include croup [16], rhinosinusitis [17], meconium aspiration syndrome [18], and transient tachypnea of the newborn (TTN) [19].

Despite growing interest in the use of NCs beyond asthma and COPD, the available literature remains heterogeneous with respect to disease conditions, patient populations, corticosteroid formulations, study designs, and reported outcomes. In addition, variations in dosing regimens, duration of therapy, and outcome assessment highlight existing gaps and inconsistencies in the evidence base, warranting a comprehensive mapping of the available literature. Based on a preliminary search conducted across several databases, we found no ongoing or completed systematic reviews or scoping reviews on this topic. Therefore, the objective of this scoping review was to explore the existing literature on the use of NCs for conditions other than asthma and COPD. Given the heterogeneity in indications, study designs, patient populations, and outcome measures, a scoping review was considered the most appropriate approach to map the available evidence.

## Review

Methods

This study followed the Preferred Reporting Items for Systematic Reviews and Meta-Analyses Extension for Scoping Reviews (PRISMA-ScR) guidelines [20]. No protocol was prospectively registered for this review. The review question, eligibility criteria, and search strategy were agreed upon by all authors before initiating the search. This scoping review focused on the use of NCs or aerosolized steroid therapy in conditions other than asthma and COPD.

Data Sources and Search Strategy

A systematic search was carried out in the PubMed and Cochrane electronic databases for articles published from the 1980s through December 31, 2025. The PubMed and Cochrane databases were selected because of their comprehensive coverage of peer-reviewed biomedical and clinical literature relevant to respiratory medicine, pharmacology, and evidence-based clinical practice. The search strategy used free-text keywords combined with Boolean operators (AND, OR, NOT) to identify relevant studies. The search strategy used in PUBMED was ((corticosteroids OR steroid OR budesonide OR beclomethasone OR flunisolide OR fluticasone) AND ((nebulised OR nebulized OR nebulizer OR nebuliser OR nebulization OR nebulisation) AND (allergy OR infection OR inflammation) NOT (asthma OR COPD OR chronic obstructive pulmonary disease OR inhaler OR rotahaler OR animal OR laboratory). The search strategy used on Cochrane to retrieve relevant literature was (Nebulizer OR nebulizer OR nebulisation) AND (steroid AND corticosteroids). No additional database filters (language, study design, or publication type) were applied during the search process to maximize sensitivity and ensure comprehensive retrieval of relevant literature. Instead, predefined eligibility criteria were applied during study selection, including the inclusion of studies published in English and interventional studies that aligned with the review objectives. The search strategy used in Cochrane to retrieve relevant literature was (Nebulizer OR nebulizer OR nebulisation) AND (steroid AND corticosteroids). A manual search of the reference lists of all included articles and relevant systematic reviews was also performed.

Study Selection and Eligibility Criteria

The articles identified using the search strategy were imported into the JBI SUMARI® software (JBI, Adelaide, SA, Australia) for deduplication. Two independent reviewers (AM and AJ) screened all titles and abstracts. Following this, the full-text articles were screened to determine their eligibility based on the inclusion criteria. Any disagreements were resolved through discussion.

Inclusion Criteria and Data Extraction

All interventional studies (randomized controlled trials, prospective randomized studies, and comparative interventional studies) conducted in children or adults evaluating the use of NCs for indications other than asthma and COPD were eligible. Given the off-label nature of the indications under review, inclusion was restricted to interventional studies to capture evidence generated through systematic clinical evaluation, thereby facilitating the mapping of potential off-label indications across different disease conditions. No restrictions were applied to the country of origin. Only studies published in English were included. Studies were excluded if they evaluated NCs exclusively in asthma or COPD, were nonhuman (animal or in vitro) studies, or were published in languages other than English. Conference abstracts, letters to the editor, correspondence, editorials, case reports, and commentaries were also excluded. Data were extracted using a standardized data-charting form developed a priori by the review team. The form was pilot-tested on a sample of included studies and refined accordingly. Data extraction was performed by one reviewer (AM) and independently verified by a second reviewer (AJ) for accuracy and completeness. Any discrepancies in the extracted data were resolved through discussion. Data were extracted from the included studies, and the key variables were defined as follows: study design referred to the methodological approach; condition studied was defined as the primary clinical diagnosis for which the NC was administered; intervention included the specific NC, dose, route of administration, frequency, and duration; comparator included placebo, active control, or standard of care; and outcomes were classified as primary and secondary. Where the studies did not distinguish between primary and secondary outcomes, all reported outcomes were extracted and presented descriptively. No imputation of missing data was performed; data that were not reported were noted as "NR" in the extraction tables. Flowcharts, bar charts, and tables were used to present data from the included studies.

Data Synthesis and Critical Appraisal

To provide a structured synthesis, the findings were categorized and presented according to clinical indications, including postoperative sore throat (POST) and airway-related outcomes, bronchiolitis and postbronchiolitic complications, TTN, cough and croup, meconium aspiration syndrome (MAS), chronic rhinosinusitis, and other respiratory conditions. Given the heterogeneity in outcome measures and reporting across studies, a narrative synthesis approach was adopted, and the findings were descriptively summarized within each clinical category without quantitative pooling or standardization of outcomes. Furthermore, because this was a scoping review intended to map the breadth of available evidence, formal risk of bias assessment, methodological quality appraisal, and certainty of evidence evaluation were not performed.

Results

Identification of Potential Studies

**Figure 1:**
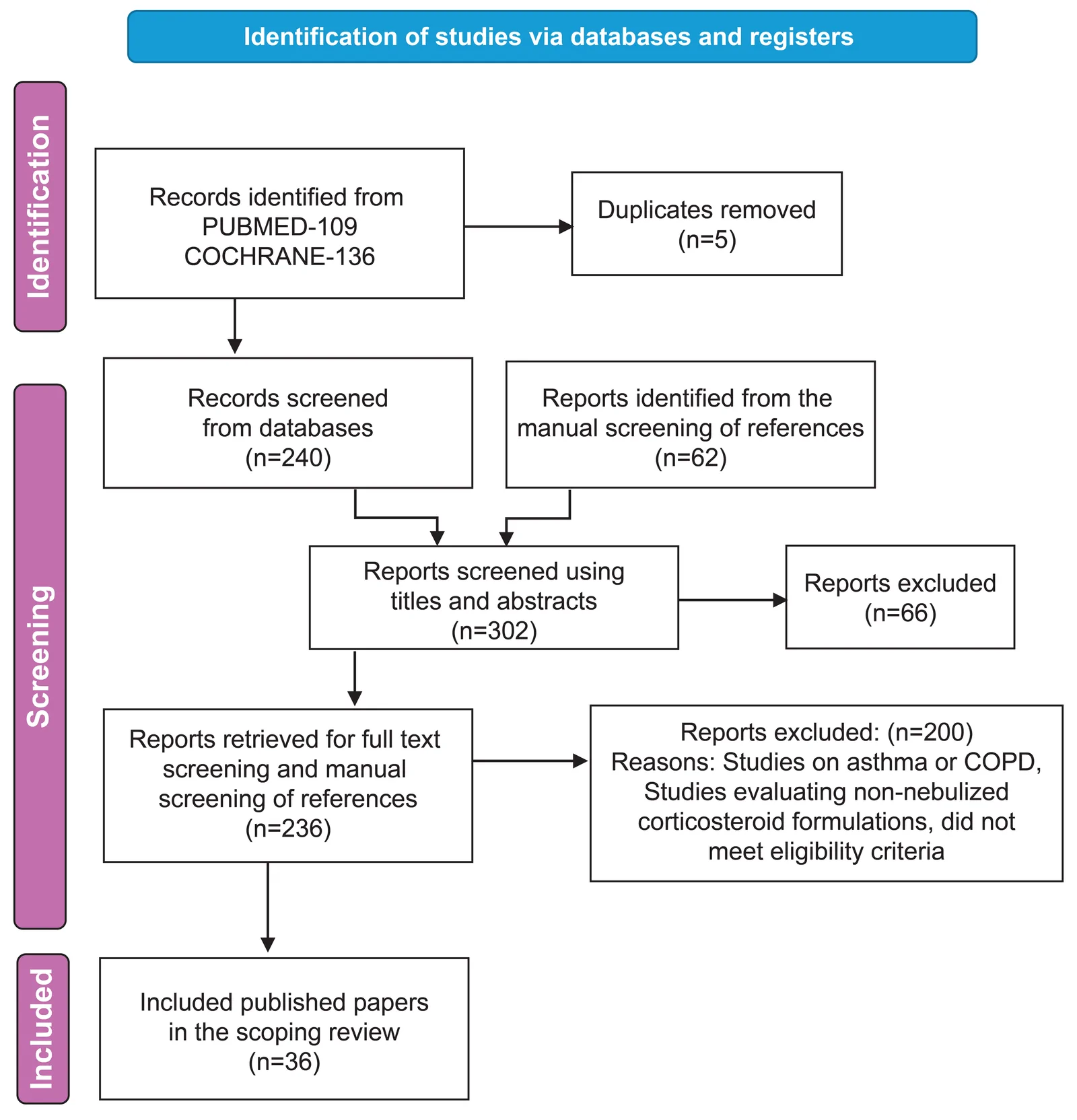
PRISMA flow diagram COPD, chronic obstructive pulmonary disease; PRISMA, Preferred Reporting Items for Systematic Reviews and Meta-Analyses

A total of 302 articles were identified through electronic database and manual searches. Following the removal of duplicates (n=5), title and abstract screening yielded 236 articles, which subsequently underwent full-text screening. Of these, 200 articles were excluded because they focused on asthma or COPD, assessed corticosteroid delivery methods other than nebulized formulations, or did not meet the eligibility criteria. Finally, 36 articles were included in this scoping review. Figure 1 presents the PRISMA flow diagram, and the characteristics of the included articles are presented in Table 1 [19,21-55].

**Table 1:** Characteristics of the included studies BO, bronchopulmonary obstruction; CRSwNP, chronic rhinosinusitis with nasal polyposis; CXR, chest X-ray; ESS, endoscopic sinus surgery; IV, intravenous; MAD, mucosal atomization device; NC, nebulized corticosteroid; NICU, neonatal intensive care unit; POST, postoperative sore throat; RSV, respiratory syncytial virus; SNOT-22, 22-item Sino-Nasal Outcome Test; TTN, transient tachypnea of the newborn; UK, United Kingdom; USA, United States of America; MAS, meconium aspiration syndrome

Study ID	Country of study	Type of study	Mean age of the participants	Condition studied	NC used, dose, and duration (N)	Control/placebo group, if any (N)	Outcomes measured
POST and airway-related outcomes							
Salama, 2016 [21]	Egypt	Randomized controlled prospective study	NC group: 37.1 years; Control group: 34.5 years	Surgical procedures in the supine position under general anesthesia with endotracheal intubation	Dexamethasone (8 mg) in 5 mL nebulization + normal saline (3 mL) (N=60); 15 minutes before intubation	Normal saline (5 mL) nebulization (N=60) for 15 minutes before general anesthesia and endotracheal intubation	Incidence of postextubation sore throat
Mehrotra, 2017 [22]	India	Randomized, double‑blind prospective study	Nebulized budesonide group: 38.93 years; Nebulized ketamine group: 34.3 years; Nebulized lidocaine group: 36.6 years; Control group: 37.2 years	POST	Nebulized budesonide (25 μg) + normal saline (1 mL) (N=30); Nebulized ketamine (50 mg) + normal saline (1 mL) (N=30); Nebulized 4% lidocaine (40 mg) + normal saline (1 mL) (N=30); 10 minutes before entering the operation theater	Nebulized distilled water (1 mL) + normal saline (1 mL) (N=30); 10 minutes before entering the operation theater	Incidence and severity of sore throat
Kumari, 2018 [23]	India	Randomized, double‑blind uncontrolled prospective study	Nebulized dexamethasone group: 37.6 years; Nebulized magnesium sulfate group: 36.9 years	Preventing POST following endotracheal intubation	Nebulized with 8 mg dexamethasone + normal saline (3 mL) (N=45); Nebulized with magnesium sulfate (50% w/v 2 mL) + normal saline (3 mL) (N=45); 30 minutes before general anesthesia	No control or placebo was used	Decreasing the incidence and severity of POST
Paul, 2019 [24]	India	Randomized, double‑blind comparative prospective study	Nebulized budesonide group: 45.0 years; Nebulized dexamethasone group: 42.3 years	Reducing POST in patients undergoing elective lumbar spine surgery in the prone position	Nebulized with 500 μg budesonide (N=60); Nebulized with 8 mg dexamethasone + normal saline (2 mL) (N=60); 20 minutes before general anesthesia	No control or placebo was used	Incidence and severity of POST
Almustafa, 2020 [25]	Jordan	Randomized controlled prospective study	NC group: 36 years; Control group: 33 years	Bougie complications encountered after sleeve gastrectomy	Nebulized with 8 mg dexamethasone (2 mL) + normal saline (3 mL) (N=40) for 30 minutes 1 hour before surgery	Nebulized with 5 mL saline (N=40)	Assessment of POST, nausea and vomiting, odynophagia, and change of voice
Bronchiolitis and postbronchiolitic complications							
Carlsen, 1988 [26]	Norway	Randomized controlled (double‑blind, placebo‑controlled) prospective study	NC group: 14.1 years; Placebo group: 17.3 years	Recurrent bronchial obstruction after acute bronchiolitis	Nebulized with 50 µg/mL beclomethasone dipropionate using a nebulizer mask; 2 mL four times/day for the first 2 weeks, 2 mL twice/day for the next 6 weeks (N=22)	Preservatives and buffers of active suspension as placebo (N=22)	Time and start of symptoms of BO, serious acute respiratory symptoms, and allergic reactions after 8 weeks
Richter, 1998 [27]	UK	Randomized controlled (double‑blind, placebo-controlled) prospective study	NC group: 0.2 years (median); Placebo group: 0.2 years (median)	Acute bronchiolitis	Nebulized budesonide 1 mg in 2 mL every 12 hours for 5 days, then 500 μg in 2 mL every 12 hours continuing for a total of 6 weeks (N=21)	0.9% saline every 12 hours for 6 weeks as nebulized placebo (N=19)	Response to therapy from baseline scores, postbronchiolitic complications
Cade, 2000 [28]	UK	Randomized controlled (double‑blind, placebo‑controlled) prospective study	NC group: 0.3 years; Placebo group: 0.3 years	Acute RSV bronchiolitis	Nebulized with 1 mg budesonide twice daily. This was given immediately after randomization and within 12 hours of admission until 14 days after being assessed as fit for hospital discharge, up to a maximum of 21 days (N=82)	Placebo (N=79)	One episode of coughing or wheezing over the 12 months after admission, length of time in the hospital, supplemental enteral and parenteral feeds or fluids, supplemental oxygen
Kajosaari, 2000 [29]	Finland	Randomized prospective study	Overall: 0.2 years	Acute RSV bronchiolitis	Inhaled budesonide 500 µg three times/day using a nebulizer for 7 days + symptomatic treatment (oxygen, bronchodilators, and/or racemic epinephrine by inhalation) (N=39); Nebulized using budesonide (500 µg) two times/day using a nebulizer for 2 months + symptomatic treatment (oxygen, bronchodilators, and/or racemic epinephrine by inhalation) (N=32)	Symptomatic treatment (oxygen, bronchodilators, and/or racemic epinephrine by inhalation) in the control group (N=38)	2- and 6-month checkups of respiratory status
Lødrup Carlsen, 2001 [30]	Norway	Randomized, double‑blind, dose response (no placebo) prospective multicenter study	High dose group: 0.7 years; Low dose group: 0.7 years	Recurrent BO	Nebulized budesonide (high dose) 1 mg (0.25 mg/mL, 2 mL twice daily) for 1 month and then 0.5 mg (0.125 mg/mL), 2 mL (twice daily) of nebulized budesonide per day during the subsequent 2 months (N=24). Nebulized budesonide (low dose), 0.2 mg (0.05 mg/mL, 2 mL twice daily) for 3 months (N=25)	No control with placebo	Number of BO episodes, time to BO, and rescue medication. Baseline, 3 months, and 12 months
Bentur, 2005 [31]	Israel	Randomized, controlled, (double‑blind) prospective study	NC group: 0.3 years; Placebo group: 0.2 years	Acute RSV bronchiolitis	Nebulized using 0.25 mg inhaled dexamethasone + epinephrine (1 mL) via facemask every 6 hours (N=10)	Nebulized using 0.5 mL 0.9% saline + epinephrine (1 mL) via facemask every 6 hours in placebo (N=10)	Respiratory rate, pulse, oxygen saturation, and clinical status
Suleman, 2023 [55]	Pakistan	Randomized controlled prospective study	NC group: 0.5 years; Control group: 0.6 years	Acute bronchiolitis	Beclomethasone dipropionate 400 µg/day via nebulization + salbutamol in 0.9% normal saline three times a day every 8 hours (N=30)	3% hypertonic normal saline nebulization three times daily at 8-hour intervals (N=30)	Proportion of patients discharged within ≤72 hours, length of hospital stay, respiratory distress severity
TTN							
Vaisbourd, 2017 [32]	Israel	Randomized, double-blind, placebo‑controlled prospective multicenter pilot study	NC group: 4 hours of age; Placebo group: 4 hours of age	TTN	Nebulized budesonide, 2 mL (1000 µg) (N=24). The first dose was given at the time of enrollment (within 6 hours of age) and the second dose, 12 hours later	0.9% normal saline solution, 2 mL (N=25) used as placebo	Respiratory rate, retractions, grunting, and nasal flaring
Elfarargy, 2020 [19]	Egypt	Randomized controlled (placebo‑controlled) prospective study	NC group: full-term neonates (>37 weeks); Placebo group: full-term neonates (>37 weeks)	TTN	Nebulized budesonide, 2 mL, 0.25 mg/mL suspension + 2 mL of 0.9% saline (N=50). The first dose was given within 6 hours of age, and the second dose, 12 hours later	2 mL of distilled sterile water used as placebo (N=50)	TTN clinical score: respiratory rate, retractions, grunting, and nasal flaring
Croup							
Husby, 1993 [33]	Denmark	Randomized controlled (double-blind, placebo-controlled) prospective study	NC group: 1.6 years (median); Placebo group: 1.1 years (median)	Mild to severe croup	Nebulized budesonide at a dose of 1000 µg (2 mL, 500 µg/mL) two times 30 minutes apart (N=20)	2 mL of 0.9% saline two times 30 minutes apart used as placebo (N=16)	Change in the croup score and overall disease severity after 2 hours
Klassen, 1994 [34]	Canada	Randomized controlled (double-blind, placebo-controlled) prospective study	NC group: 1.8 years; Placebo group: 2.2 years	Mild to severe croup	Nebulized budesonide 2 mg (4 mL) single dose (N=27)	4 mL of 0.9% normal saline was used as placebo (N=27)	Croup score, general condition, and duration of stay in the emergency department
Geelhoed, 1995 [35]	Australia	Randomized controlled (double-blind, placebo-controlled) prospective study	Budesonide group: 2.7 years; Dexamethasone group: 2.9 years; Placebo group: 2.5 years	Mild to severe croup	Oral dexamethasone BP (0.6 mg/kg, maximum 12 mg) and 4 mL saline nebulization (N=27). Nebulized budesonide and an equivalent volume of oral placebo (N=23)	Oral placebo and nebulized saline (4 mL) (N=30)	Croup score and duration of hospitalization stay
Fitzgerald, 1996 [36]	Australia	Randomized controlled (double-blind) multicenter prospective study	Budesonide group: 20.9 years; Adrenaline group: 24.9 years	Mild to severe croup	Nebulized budesonide (2 mg/4 mL) (N=35)	L-adrenaline (4 mg/mL) (N=31)	Croup scores, oxygen saturation, duration of hospitalization, subsequent systemic steroid, and adverse events 24 hours after nebulization.
Johnson, 1996 [37]	Canada	Randomized controlled (double-blind, placebo-controlled) prospective study	NC group: 1.2 years (median); Placebo group: 1.4 years (median)	Moderate croup	The treatment group received 10 mg (<8 kg body weight), 15 mg (8-12 kg), or 20 mg (>12 kg) of parenteral dexamethasone sodium phosphate solution (10 mg/mL) with a nebulizer mixed with normal saline to make 4 mL (N=28)	The control group received normal saline (N=27)	Croup score
Klassen, 1996 [38]	Canada	Randomized controlled (double-blind, placebo-controlled) prospective study	0.25-5 years (range)	Mild to severe croup	The treatment group received 0.6 mg/kg oral dexamethasone and was randomly assigned to receive 4 mL (2 mg) of budesonide solution (n=25) by nebulizer	0.6 mg/kg oral dexamethasone and randomly assigned to receive 4 mL of 0.9% saline solution (n=25) by nebulizer	Proportion of patients with a decrease in the croup score by 2 points within 4 hours of treatment
Godden, 1997 [39]	UK	Randomized controlled (double-blind, placebo-controlled) prospective study	NC group: 2.9 years; Placebo group: 3.1 years	Mild to severe croup	Initial dose with nebulized budesonide 2 mg (4 mL) followed by budesonide 1 mg every 12 hours (N=47)	2 mL of normal saline every 12 hours was used as placebo (N=42)	Croup score, length of hospital stays
Roberts, 1999 [40]	Australia	Randomized controlled (double-blind, placebo-controlled) prospective study	NC group: 2.3 years; Placebo group: 2.2 years	Mild to severe croup	Nebulized budesonide, 2 mg/4 mLevery 12 hours (N=42)	4 mL of placebo (identical formulation without budesonide) every 12 hours (N=40)	Croup score, time to clinical improvement
Johnson, 1998 [41]	Canada	Randomized controlled (double-blind, placebo-controlled) prospective study	Overall: 2 years	Mild to severe croup	Nebulized budesonide (4 mg) + 0.5 mL of 2.25% racepinephrine + normal saline (N=48) Intramuscular dexamethasone (0.6 mg/kg of body weight + 0.5 mL of 2.25% racepinephrine + normal saline (N=47))	0.5 mL of 2.25% racepinephrine + normal saline was used as placebo suspension (N=49)	Hospitalization rates, croup score, respiratory rate, heart rate
Luria, 2001 [42]	USA	Randomized controlled (double-blind, placebo-controlled) prospective study	Oral dexamethasone group: 2.3 years; Nebulized dexamethasone group: 2.5 years; Placebo group: 2.1 years	Mild to severe croup	Oral dexamethasone 0.6 mg/kg + nebulized placebo (N=85). Nebulized dexamethasone sodium phosphate 160 µg + oral placebo (N=91)	Oral and nebulized placebo (1 mg/mL solution of dexamethasone intensol) (N=88)	Resolution of croup symptoms
MAS							
Basu, 2007 [44]	India	Randomized controlled prospective study	Budesonide group: 12 hours of age; Methylprednisolone group: 12 hours of age; Control group: 12 hours of age	MAS	Nebulized budesonide 50 µg + normal saline 2.5 mL every 12 hours for 7 days (started 24-36 hours after birth) (N=32)	Systemic methylprednisolone 0.5 mg/kg/day IV every 12 hours for 7 days (started 24–36 hours after birth) (N=34) Control group (N=33)	Duration of respiratory distress, oxygen requirements, duration of hospital stays, the requirement of IV fluids, starting of feeding, achievement of full feeding, and CXR clearance
Tripathi, 2007 [45]	India	Randomized controlled (double-blind) prospective study	Methylprednisolone group: Newborn babies (>2 kg weight); Budesonide group: Newborn babies (>2 kg weight); Control group: Newborn babies (>2 kg weight)	MAS	Nebulized budesonide 50 µg in 2.5 mL normal saline by nebulization every 12 hours for 7 days (N=17)	Systemic methylprednisolone 0.5 mg/kg/day IV every 12 hours for 7 days (N=17). The control group received standard care (N=17)	Duration of stay, survival, duration of oxygen dependence, duration of X-ray clearance, feeding, and discontinuation of IV fluids
Rangaraj, 2015 [46]	India	Randomized controlled prospective study	Overall: Newborn	MAS	Nebulized budesonide 50 µg in 2.5 mL of normal saline every 12 hours from the second day of life till 7 days or clinical recovery (whichever was earlier) (N=20)	Nebulized normal saline in control (N=20)	Duration of respiratory distress, duration of oxygen dependency, duration of hospital stays, time until full feeding, and need for mechanical ventilation
Garg, 2016 [47]	India	Randomized controlled prospective study	Overall: Newborn	MAS	Nebulized budesonide 0.5 mg with normal saline (2.5 mL) within 2 hours of birth and the second dose at 12 hours after birth via nebulizer system (N=39)	Nebulized normal saline (2.5 mL) within 2 hours and at 12 hours after birth via nebulizer system (N=39)	Respiratory score (Downes’ score), requirement (dependence) of oxygen, and duration of NICU stay
Rana, 2018 [48]	India	Randomized controlled prospective study	Overall: Newborn	MAS	Systemic methylprednisolone 0.5 mg/kg/day in 12 hourly intervals + nebulized budesonide 0.5 mg in 2.5 mL of normal saline every 12 hours for 7 days + conventional management (N=137)	Systemic normal saline and nebulization with 3% normal saline in placebo (N=136)	Respiratory score (Downes’ score), requirement (dependence) of oxygen, time until resolution of respiratory distress, days on mechanical ventilation, duration of hospitalization, and long-term complications
Chronic rhinosinusitis (associated with or without endoscopic surgery)							
Thamboo, 2014 [49]	Canada	Randomized controlled prospective pilot study	Overall: >20 years	Chronic rhinosinusitis without nasal polyp	1 mg of budesonide twice daily via the MAD syringe (N=10)	1 mg of budesonide twice daily in 120 mL of saline solution in the impregnated nasal saline irrigation group (N=10)	Sino nasal outcome test
Wang, 2015 [50]	China	Randomized controlled (double‑blind, placebo‑controlled) prospective study	NC group: 48.2 years; Placebo group: 44.0 years	Chronic rhinosinusitis with nasal polyps	1 mg of budesonide via trans nasal nebulization twice daily for 14 days with nebulizer (N=30)	Saline solution used as placebo (N=30)	Reduction in nasal polyp size and treatment of several markers of inflammation and nasal tissue remodeling
Neubauer, 2016 [51]	USA	Randomized controlled prospective study	Overall: 44.3 years	CRSwNP with ESS	Nebulized budesonide (0.5 mg/2 mL; one-half instilled into each nostril in the upright position) via a MAD atomizer two times per day (N=11). Nebulized budesonide (0.5 mg/2 mL; one-half instilled into each nostril in the vertex-to-floor position) via a MAD atomizer two times per day (N=11)	1. Fluticasone nasal spray with two 50 µg sprays to each nostril two times per day (N=10)	SNOT‑22 and Lund–Kennedy scores at 6 months
Dai, 2017 [52]	China	Randomized controlled prospective single-blind study	NC group: 43.2 years; Control group: 42.4 years	Allergic fungal rhinosinusitis	1 mg of budesonide; transnasal nebulization twice daily for 2 weeks; once a day for the next 2 weeks; once every 2 days for the next 4 weeks; twice per week for the next 8 weeks; and then once a week to maintain treatment (N=15)	Budesonide nasal pump sprays (N=15)	Nasal symptoms, Lund-Mackay scores, and Kupferberg grades
Zhang, 2019 [53]	China	Randomized controlled (head-to-head) prospective study	Nebulized budesonide group: 42.3 years; Oral methylprednisolone group: 43.4 years; Budesonide nasal spray group: 43.1 years	Chronic rhinosinusitis with nasal polyps	1 mg/2 mL budesonide inhalation suspension via transnasal nebulization twice daily for 2 weeks (N=30)	Oral methylprednisolone 24 mg once a day for 2 weeks (N=26); Budesonide nasal spray 256 μg twice a day for 2 weeks (N=29)	Relief of symptoms, reduction in polyp size, overall control of mucosal inflammation, and tissue remodeling
Additional respiratory conditions							
Clavenna, 2014 [54]	Italy	Randomized controlled (double‑blind, placebo‑controlled) prospective study	NC group: 2.1 years; Placebo group: 2.1 years	Viral wheezing	400 µg beclomethasone as 1 mL of suspension in 1 mL of saline administered through a pneumatic nebulizer twice daily for 10 days (N=264)	Placebo (N=261)	Incidence of viral wheezing and respiratory symptoms during the 10-day treatment period
Ogawa, 2015 [43]	Japan	Randomized controlled (single-blind, parallel-controlled) prospective study	21-75 years (range)	Cough-related laryngeal sensations	Nebulized amphotericin B 2.5 mg (N=20). Nebulized budesonide 0.5 mg (N=20)	No placebo used	Cough-related laryngeal sensation questionnaire

**Figure 2:**
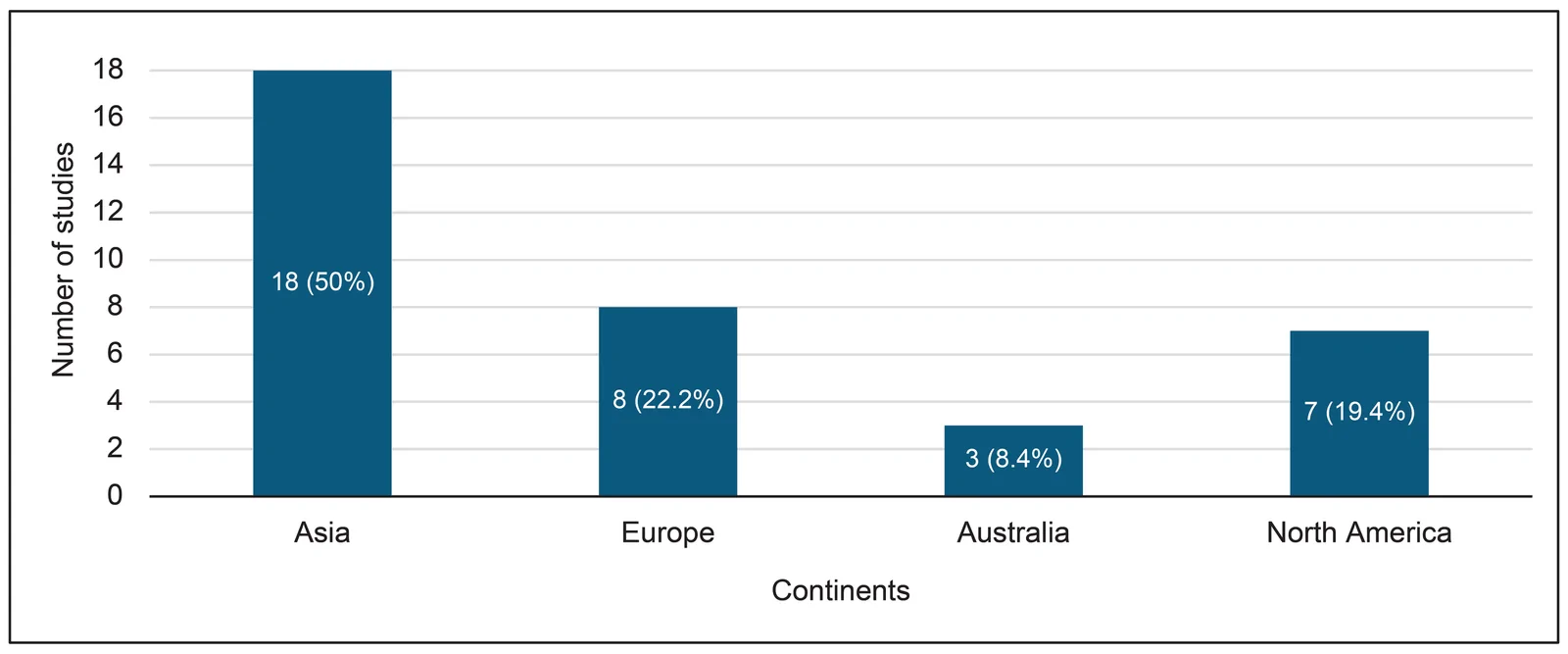
Number of studies by continent

Most of the studies included in this scoping review were conducted in Asia (n=18, 50%), followed by Europe (n=8, 22.2%), North America (n=7, 19.4%), and Australia (n=3, 8.4%) (Figure 2). The studies were prospective interventional studies. The majority were double-blind, placebo-controlled studies. The included studies enrolled a total of 3,415 patients (range: 20-525; median: 78). Furthermore, the studies encompassed a broad age range, including infants, children, adolescents, and adults, with reported ages ranging from <1 year to 48.2 years. Treatment duration varied substantially across the included studies, ranging from single-dose interventions to long-term regimens extending up to six months (Table 1).

Descriptions of the Use of Nebulized Corticosteroids in Conditions Other Than Asthma and Chronic Obstructive Pulmonary Disease in Interventional Studies

**Figure 3:**
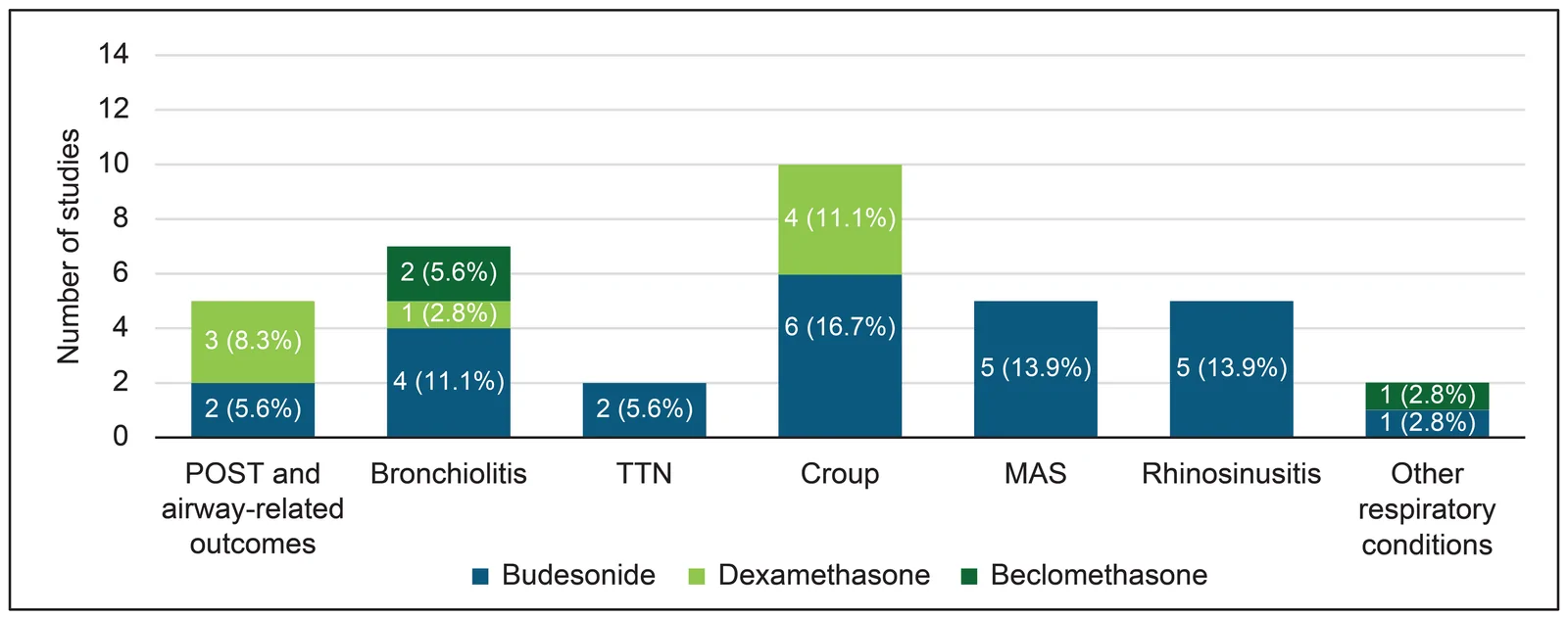
Number of studies showing steroid use across different indications MAS, meconium aspiration syndrome; POST, postoperative sore throat; TTN, transient tachypnea of the newborn

The characteristics of the conditions (other than asthma and COPD) for which NCs are used are shown in Figure 3. In this review, 24 (66.7%) of the included studies were conducted in children, including infants and neonates, while 12 (33.3%) were conducted in adults. The greatest number of studies focused on croup (n=10, 27.8%), followed by bronchiolitis (n=7, 19.5%); other studies evaluated POST and airway-related outcomes (n=5, 13.9%), chronic rhinosinusitis (n=5, 13.9%), MAS (n=5, 13.9%), and TTN (n=2, 5.6%). Other indications included viral wheezing (n=1, 2.8%) and cough-related laryngeal sensations (n=1, 2.8%). Budesonide was the most frequently studied corticosteroid in these conditions (n=25, 69.4% of studies), followed by dexamethasone (n=8, 22.2%) and beclomethasone (n=3, 8.3%) (Figure 3).

Descriptions of the Outcomes Measured

The key findings from the included studies are presented in Table 2 [19,21-55].

**Table 2:** Key findings from the included studies in this scoping review CRS, chronic rhinosinusitis; CRSwNP, chronic rhinosinusitis with nasal polyposis; ECRSwNP, eosinophilic chronic rhinosinusitis with nasal polyps; ESS, endoscopic sinus surgery; IgE, immunoglobulin E; IL, interleukin; INSI, impregnated nasal saline irrigation; IV, intravenous; MAD, mucosal atomization device; MAS, meconium aspiration syndrome; MMP, matrix metalloproteinase; NC, nebulized corticosteroids; NR, not reported; POST, postoperative sore throat; RDAI, Respiratory Distress Assessment Instrument; RSV, respiratory syncytial virus; SMIT, sensation of mucus in the throat; SNOT-22, 22-item Sino-Nasal Outcome Test; TH2, T-helper 2; TIMP, tissue inhibitors of metalloproteinase; TNSS, total nasal symptom score; TTN, transient tachypnea of the newborn; URTI, upper respiratory tract infection; VF, ventricular fibrillation

Study ID	Primary findings	Secondary findings	Conclusion
POST and airway-related outcomes			
Salama, 2016 [21]	POST: The incidence and severity of POST were significantly lower in the dexamethasone-treated group than in the saline-treated group at 0, 2, 4, 8, and 12 hours (p<0.05) with no significant difference at 24 hours after extubation (p=0.513)	No adverse effects were encountered in the groups.	Preoperative nebulization with dexamethasone (8 mg) reduces the incidence and severity of POST in patients receiving general anesthesia with endotracheal intubation.
Mehrotra, 2017 [22]	Sore throat: Incidence decreased from 48.33% at 1 hour to 21.66% at 48 hours. At 48 hours, budesonide was most (significantly) effective, followed by ketamine and lignocaine. Cough and hoarseness: Incidence of cough decreased from 22.5% to 10% and hoarseness from 35% to 20% (from 1 hour to 48 hours).	NR	Budesonide was less effective in reducing sore throat compared with ketamine and lignocaine at 1 hour and 24 hours. At 48 hours, budesonide showed the least sore throat incidence compared with the other corticosteroids. Lignocaine showed the lowest incidence of cough at 1 hour and 24 hours compared with the other groups. Long-term outcomes were better with budesonide (48 hours).
Kumari, 2018 [23]	Pain at rest: Lower with dexamethasone vs. magnesium sulfate at 12 hours (3% vs. 30%); comparable at 24 hours. Pain on swallowing: Lower with dexamethasone vs. magnesium sulfate at 12 hours (3% vs. 28%); comparable at 24 hours. Hoarseness: Absent with dexamethasone vs. 8% with magnesium sulfate at 12 hours; none in either group at 24 hours (comparable). Cough: The incidence of cough was comparable between the groups at all time intervals.	NR	Preoperative dexamethasone nebulization just before the induction of anesthesia is an effective method of reducing the incidence and severity of POST following endotracheal intubation.
Paul, 2019 [24]	Sore throat: Significantly higher with budesonide vs. dexamethasone at 24 hours (38.7% vs. 5%). Hoarseness: Significantly higher with budesonide vs. dexamethasone at 24 hours (46.7% vs. 0%). Cough: Comparable between budesonide and dexamethasone groups at 24 hours (5% vs. 0%).	NR	Single prophylactic preinduction nebulization, when given to patients undergoing elective lumbar spine surgery in a prone position, with dexamethasone is better than budesonide in preventing POST.
Almustafa, 2020 [25]	Pain score of sore throat: Significantly lower with dexamethasone vs. saline at 24 hours (0.03 vs. 0.7).	Postoperative nausea and vomiting were observed in 85% of the patients from the dexamethasone group and 90% of the patients from the saline group. Odynophagia was observed in 2.5% of the patients from the dexamethasone group and 7.5% of the patients from the saline group. Change of voice was observed in 10% of the patients from the dexamethasone group and 47.5% of the patients from the saline group.	Preoperative nebulized dexamethasone was found to be an effective measure in reducing bougie insertion complications in laparoscopic sleeve gastrectomy.
Bronchiolitis and postbronchiolitic complications			
Carlsen, 1988 [26]	Time until the first episode of bronchopulmonary obstruction: Significantly longer with beclomethasone vs. placebo (7 vs. 4 weeks). Episodes of bronchopulmonary obstruction: Significantly lower with beclomethasone vs. placebo (4.5 vs. 9.0). Total obstructive score: Significantly lower with beclomethasone vs. placebo (12.0 vs. 19.5). Symptomatic respiratory illness: Significantly fewer episodes with beclomethasone vs. placebo (6.5 vs. 10). Allergic reaction: The total IgE levels were comparable between beclomethasone and placebo (15.5 kU/l vs. 11 kU/l).	NR	The beclomethasone group was significantly more protected from developing acute respiratory illness or bronchopulmonary obstruction than the placebo group.
Richter, 1998 [27]	Wheezing: Prevalence of wheezing after treatment completion was comparable between the budesonide and placebo group (75% vs. 79%). Cough and wheeze scores: Median score for coughing and wheezing was comparable between budesonide and placebo (10.0 vs. 10.0). Readmission: Readmissions were significantly higher with budesonide vs. placebo (50% vs. 10.5%).	NR	No significant differences were found between the budesonide and placebo groups in reducing the symptoms of acute bronchiolitis or preventing postbronchiolitis wheezing.
Cade, 2000 [28]	Time to become asymptomatic for 48 hours: The hazard ratio for time to become asymptomatic (defined as absence of cough and wheeze for 48 hours) in the budesonide group compared with placebo was 1.41, indicating a trend toward faster symptom resolution with budesonide. Coughing/wheezing episodes: Mean number of episodes of coughing/wheezing after discharge was comparable between budesonide and placebo (17.0 vs. 17.1). Readmission: Mean number of infant readmissions for respiratory problems was comparable between budesonide and placebo (13.0 vs. 14.0).	NR	No significant difference in any of the outcomes. There are no short- or long‑term clinical benefits from the administration of NCs in the acute phase of RSV bronchiolitis.
Kajosaari, 2000 [29]	Respiratory status: Two years after infection, the proportion of patients requiring asthma medication was lowest with nebulized budesonide (12%), followed by inhaled budesonide via nebulizer (18%), and highest with symptomatic treatment (37%).	NR	NCs during and after the acute phase of infant RSV bronchiolitis may have a beneficial effect on subsequent bronchial wheezing tendencies.
Lødrup Carlsen, 2001 [30]	Time until the first episode of bronchial obstruction: The time to first episode of bronchial obstruction was comparable between high-dose budesonide and low-dose budesonide (31 days vs. 41.3 days). Episodes of bronchial obstruction: Mean number of episodes of bronchial obstruction at 12 months was comparable between high-dose budesonide and low-dose budesonide (10.4 vs. 12.6). Hospitalizations: Mean number of hospitalizations at 12 months was comparable between high-dose budesonide and low-dose budesonide (3.1 vs. 4.2).	Adverse effects: Serious adverse events were reported in 24.4% of the patients taking budesonide.	There was no significant dose‑dependent beneficial effect of 3 months of treatment with nebulized budesonide. However, there was a tendency for fewer hospitalizations and less use of antiasthmatic medication in children treated with the highest doses of nebulized budesonide.
Bentur, 2005 [31]	Recurrent hospitalizations: Recurrent hospitalization was comparable between dexamethasone and placebo (41.35% vs. 43%). Recurrent wheeze: Recurrent wheeze was comparable between dexamethasone and placebo (51.7% vs. 53.1%). Length of hospitalization: Length of hospitalization in prematurely born infants was significantly less in dexamethasone than placebo (6.5 days vs. 9.1 days). For full-term infants, the length of hospitalization was comparable (5.2 days vs. 5.5 days).	NR	No statistically significant differences concerning clinical score, oxygen saturation, or IV fluid requirement between the groups were noted.
Suleman, 2023 [55]	RDAI score: RDAI scores were similar at baseline between the NC (beclomethasone dipropionate + salbutamol) and 3% saline groups (9.52±1.16 vs. 9.61±1.41) but were significantly higher in the NC group at 72 hours (3.20±1.40 vs. 1.80±0.36). Length of hospital stay: Mean length of hospital stay was significantly longer in the NC (beclomethasone dipropionate + salbutamol) group than the 3% saline group (3.58±0.82 days vs. 2.76±0.51 days). Discharge within ≤72 hours: A significantly lower proportion of infants in the NC (beclomethasone dipropionate + salbutamol) group were discharged within ≤3 days compared with the 3% saline group (63.33% vs. 90%).	NR	Nebulization with 3% normal saline was significantly more effective than steroid nebulization in alleviating symptoms and reducing the severity of bronchiolitis.
TTN			
Vaisbourd, 2017 [32]	TTN: TTN clinical score at 48 hours was comparable between budesonide and placebo (0.5 vs. 0.6). Respiratory support: Respiratory support required at 48 hours was comparable between budesonide and placebo (37% vs. 44% of patients).	Time to spontaneous unsupported breathing: The time to spontaneous unsupported breathing was 67.4 hours in the budesonide group, which was comparable with that of the placebo group (75.2 hours). Length of hospitalization: The length of stay was 9.9 days in the budesonide group, which was comparable with that of the placebo group (12.5 days).	Unable to detect a beneficial effect of early administration of steroids on the clinical course of TTN in late preterm and term infants.
Elfarargy, 2020 [19]	Respiratory distress: Mean Down score was significantly less with budesonide than with placebo (3 vs. 4.5). TTN: Mean TTN clinical score was significantly lower with budesonide than with placebo (1.8 vs 2.9). Length of hospitalization: Length of hospitalization was significantly lower with budesonide than with placebo (4.7 days vs. 6.2 days).	NR	Administration of budesonide showed improvement in TTN cases.
Croup			
Husby, 1993 [33]	Croup score: Change in total croup score was significantly greater with budesonide than with placebo (+4 vs. 0), indicating improvement. Stridor: Change in stridor was significantly greater with budesonide than with placebo (+1 vs. 0). Disease severity: Disease severity improved significantly with budesonide than with placebo (+25 vs. 0).	Adverse effects: No side effects were observed.	Nebulized budesonide can be used as a safe and effective alternate treatment in children and infants with moderate to severe croup.
Klassen, 1994 [34]	Croup score: Reduced from a median value of 4 at baseline to 1 at follow-up in the budesonide group and from 4 at baseline to 3 at the follow-up in the placebo group. Change in patients’ condition: The global assessment of change favored patients assigned to budesonide treatment over those on placebo.	NR	Nebulized budesonide leads to prompt clinical improvement in children who come to the emergency department with mild-to-moderate croup.
Geelhoed, 1995 [35]	Croup score: Baseline scores were 3.7 (budesonide) and 3.8 (placebo and dexamethasone); reduction to ≤1 occurred fastest with budesonide (1 hour), followed by dexamethasone (2 hours) and placebo (8 hours). Length of hospitalization: Length of hospitalization was 13 days in the budesonide group, 20 days in the placebo group, and 12 days in the dexamethasone group. Readmission: Readmission was comparable across the groups, with rates of 7.4% (budesonide), 6.6% (placebo), and 0% (dexamethasone).	NR	Oral dexamethasone and nebulized budesonide are both effective in reducing the symptoms and duration of hospitalization in children with croup.
Fitzgerald, 1996 [36]	Croup score: The croup scores in the budesonide and L-adrenaline groups were comparable. Length of hospitalization: The length of hospitalization in the budesonide and L-adrenaline groups was comparable.	Adverse events: The adverse events in the budesonide and L-adrenaline groups were comparable.	There were no significant differences in the efficacy and safety between the treatment groups.
Johnson, 1996 [37]	Croup score: The croup score reduced from a median of 4 at baseline to 2 at 4 hours in the dexamethasone group and from 4 at baseline to 3 at 4 hours in the placebo group. Readmission: Readmission was comparable between dexamethasone and placebo (22.2% vs. 16.6%) at 24 hours.	Adverse effects: Two hospitalizations in the dexamethasone group due to severe inspiratory airway obstruction requiring prolonged intubation.	Treatment of moderate croup with nebulized dexamethasone results in clinical improvement within 4 hours. However, due to infectious complications, the study results do not recommend that patients with croup be treated with nebulized dexamethasone.
Klassen, 1996 [38]	Clinical response: 84% of the patients in the budesonide group had clinically important responses, compared with 56% of the patients in the placebo group.	NR	Nebulized budesonide improved respiratory symptoms in children with mild-to-moderate croup, providing additional benefit beyond oral dexamethasone alone.
Godden, 1997 [39]	Croup score: Croup score was significantly lower in the budesonide group than in the placebo group (1.16 vs. 3.23) at 24 hours.	NR	Nebulized budesonide is an effective treatment for children admitted to the hospital with a clinical diagnosis of croup.
Roberts, 1999 [40]	Stridor: The prevalence of stridor at day 3 after discharge was 3% in the budesonide group and 16% in the placebo group. Cough: The prevalence of cough on day 3 after discharge was 41% in the budesonide group and 34% in the placebo group. Hoarseness: The prevalence of hoarseness on day 3 after discharge was 44% in the budesonide group and 31% in the placebo group.	Adverse effects: 21.4% of the patients reported adverse effects (exacerbation in symptoms, emotional distress, vomiting, eye irritation) in the budesonide group and 40% (exacerbation in symptoms, emotional distress, vomiting, rash, eye irritation, irritated tongue) in the placebo group.	Twelve-hourly dosing with nebulized budesonide significantly improved the symptoms of croup as well as decreased relapse rates when compared with the placebo.
Johnson, 1998 [41]	Croup score: The change in the mean croup score was -2 in the budesonide group, -2.9 in the dexamethasone group, and -1.3 in the placebo group.	Adverse effects: None of the patients had gastrointestinal bleeding or bacterial tracheitis.	Treatment with intramuscular dexamethasone or nebulized budesonide resulted in more rapid clinical improvement than the administration of placebo.
Luria, 2001 [42]	Viral isolates: Viral isolates were comparable across oral dexamethasone, nebulized dexamethasone, and placebo groups for parainfluenza (40%, 35.1%, 38.6%), influenza A (2.3%, 6.5%, 1.1%), and RSV (2.3%, 1.1%, 1.1%).	Treatment failure: Treatment failure rates differed significantly across groups, with treatment failure reported in 3.5% of patients receiving oral dexamethasone, 11.3% receiving placebo, and 13.1% receiving nebulized dexamethasone.	Patients with mild croup on oral dexamethasone are less likely to seek subsequent care and show more rapid clinical improvement compared with those on nebulized dexamethasone or placebo.
MAS			
Basu, 2007 [44]	X-ray clearance: X-ray clearance of chest was achieved significantly faster with budesonide and methylprednisolone vs. control (6.56 and 6.65 vs. 15.8 days); steroid groups had comparable clearance. Respiratory distress: Time to achieve a score of 0 was significantly shorter with budesonide and methylprednisolone vs. control (4.59 and 4.41 vs. 7.7 days), with comparable results between the steroid groups. Full feed achieved: Time to full feeds was significantly shorter with budesonide and methylprednisolone vs. control (6.41 and 6.56 vs. 13.9 days), with comparable results between the steroid groups. Length of hospitalization: The length of hospitalization was 10.6 days in the budesonide group, 10.8 days in the methylprednisolone group, and 18.0 days in the control group.	Adverse effects: Development of complications (meningitis, hypotension, anemia, hypoglycemia, hypocalcemia, and respiratory arrest) was similar in the budesonide, methylprednisolone, and control groups.	Steroids are effective in the management of MAS, and the route of administration does not affect the efficacy.
Tripathi, 2007 [45]	X-ray clearance: Time to clearance was comparable between budesonide and methylprednisolone (5.1 vs. 5.4 days) and significantly shorter than control (7.7 days). Length of hospitalization: Length of hospitalization was comparable between budesonide and methylprednisolone (12.1 vs. 13.2 days) and significantly shorter than control (19.5 days).	Adverse effects: Development of complications (meningitis, fungal infection, loose motions, oral thrush, positive blood culture) was similar in the budesonide, methylprednisolone, and control groups.	Steroids were found to be beneficial as a treatment modality in MAS. Both nebulized and systemic steroids are equally efficacious.
Rangaraj, 2015 [46]	Respiratory distress: The duration of respiratory distress was significantly shorter with budesonide vs. control (2.6 vs. 5.2 days). Full feed achieved: Time to full feed was significantly shorter with budesonide vs. control (3.7 vs. 8.7 days). Length of hospitalization: Length of hospitalization was significantly shorter with budesonide vs. control (7.5 vs. 10.4 days).	Adverse effects: Development of complications (meningitis, sepsis without meningitis, hypotension, pneumothorax, seizures, hypoglycemia, hyperglycemia, hyperbilirubinemia, mortality) was similar in the budesonide and control groups.	Nebulized budesonide improves the short-term respiratory outcome and is relatively safe and effective compared with control.
Garg, 2016 [47]	Respiratory distress: The respiratory distress score (Downes’ score) was significantly lower with budesonide vs. control (1.8 vs. 2.9). Normalization of respiratory rate: Significantly more patients achieved normalization of respiratory rate within 1-3 days with budesonide vs. control (92% vs. 8%). Duration of oxygen requirement: Duration of oxygen requirement was shorter with budesonide, with 82% requiring oxygen for 1-2 days vs. 3-6 days in the control group.	Adverse effects: No short-term adverse effects were reported with budesonide use.	Budesonide nebulization in meconium aspiration results in a significant early improvement in the general condition of the newborn with lesser oxygen requirement.
Rana, 2018 [48]	Respiratory distress: The respiratory distress score (Downes’ score) was significantly lower with steroids vs. control (0.0 vs. 1.0). Duration of oxygen requirement: Oxygen dependency was significantly shorter with steroids vs. control (3 vs. 4 days). Length of hospitalization: Length of hospitalization was significantly shorter with steroids vs. control (8 vs. 9 days).	Adverse effects: 5.8% of the patients in the steroid group developed long-term complications (bronchopulmonary dysplasia, cerebral palsy), which was significantly lower than that in the control group (19.7%).	A remarkable and statistically significant improvement was observed in the clinical course of neonates receiving IV and nebulized steroids; this included a reduction in long-term complications, including bronchopulmonary dysplasia and cerebral palsy.
CRS (associated with or without endoscopic surgery)			
Thamboo, 2014 [49]	SNOT-22 scores: SNOT-22 scores significantly improved from baseline to 60 days in the budesonide via MAD group, while no significant improvement was observed in the budesonide via INSI group over time.	Adverse effects: About 10% of patients experienced nosebleeds in the budesonide via MAD group, and 30% experienced discomfort in the budesonide via INSI group.	No significant difference between the groups. However, patients with CRS who are administered budesonide via MAD may experience significant symptomatic improvement compared with those using budesonide-impregnated nasal lavage.
Wang, 2015 [50]	Nasal polyp size: Reduction in nasal polyp size on day 14 was significantly greater in the budesonide group than the placebo group. TNSS: Nasal symptom scores improved significantly more at the end of treatment with budesonide than placebo.	Reduction in eosinophil infiltration and TH2 cell frequency and increased natural regulatory T-cell and type 1 regulatory T-‍cell frequencies in the budesonide group. Significant decrease in the indices of remodeling, including albumin, MMP-2, MMP-7, MMP-8, and MMP-9 in the budesonide group. Significant increase in collagen deposition and TIMP-1, TIMP-2, and TIMP-4 levels in the budesonide group.	Short-term budesonide transnasal nebulization is an effective and safe treatment option in patients with eosinophilic CRSwNP, achieving clinical improvement by regulating remodeling, cytokine expression, and T-‍cell subset distribution.
Neubauer, 2016 [51]	SNOT-22 scores: The SNOT-22 score at 6 months was significantly lower with budesonide MAD vs. budesonide VF and vs. fluticasone (10.29 vs. 15.88 vs. 31.5). Lund–Kennedy scores Lund–Kennedy scores at 6 months were significantly lower with budesonide MAD vs. budesonide VF and fluticasone (0.50 vs. 1.1 and 2.6).	Adverse effects: No adverse effects were reported with budesonide or fluticasone use.	Patients treated with budesonide after ESS for CRSwNP had greater improvement in the SNOT-22 and Lund–Kennedy scores compared with fluticasone at 6 months. The data support the use of budesonide, particularly with a MAD, over fluticasone for patients with CRSwNP after ESS.
Dai, 2017 [52]	TNSS: Improvement in TNSS from baseline to 24 weeks was comparable between nebulized and topical nasal budesonide groups (5.67 vs 6.0). Kupferberg grades: Improvement in Kupferberg grades from baseline to 24 weeks was comparable between nebulized and topical nasal budesonide groups (0.0 vs. 0.2). Recurrence of allergic fungal rhinosinusitis: No patients in the nebulized budesonide group developed recurrence, while 26.6% of those in the topical nasal budesonide group developed recurrence.	NR	Nebulized budesonide is an effective and safe treatment for patients with allergic fungal rhinosinusitis following ESS, as evidenced by the reduced recurrence rate observed in the budesonide transnasal nebulization group relative to the topical nasal steroids group.
Zhang, 2019 [53]	TNSS: TNSS improved significantly in the methylprednisolone, nebulized budesonide, and nasal spray budesonide groups, with greater improvement in the methylprednisolone and nebulized budesonide groups vs. the budesonide nasal spray group. Nasal polyp score: Nasal polyp score improved significantly with methylprednisolone (from 4.9 to 2.8) and nebulized budesonide (from 4.6 to 3.3), with no significant improvement observed with budesonide nasal spray.	Significant reductions in eosinophils, TH2 cells, eosinophil cationic protein, IL-5, and expression of MMP-2 and -9 were noted in the methylprednisolone and nebulized budesonide groups as compared with those in the nasal spray budesonide group. Significant increases in type 1 regulatory T cells, IL-10, transforming growth factor-β, and TIMP-1 and -2 were noted in the nasal polyps in the methylprednisolone and nebulized budesonide groups as compared with those in the nasal spray budesonide group.	A short course of budesonide inhalation suspension transnasal nebulization is more efficacious than budesonide nasal spray in the management of ECRSwNP and is safer than oral methylprednisolone with regard to the hypothalamic-pituitary-adrenal axis function.
Additional respiratory conditions			
Clavenna, 2014 [54]	Wheeze: Wheezing episodes were comparable between beclomethasone and placebo (6.8% vs. 11.1%). URTI: Both groups had comparable numbers of patients with URTI at visit 2 (40.9% in the beclomethasone group and 44.1% in the placebo group).	Adverse effects: Adverse effects (hoarseness, diarrhea, skin rash, vomiting, and candidiasis) were comparable between beclomethasone and placebo (38% vs. 39%).	Steroids are not effective in preventing the recurrence of viral wheezing. Moreover, no benefits were found in reducing symptoms of respiratory tract infections.
Ogawa, 2015 [43]	Cough-related laryngeal sensation: Throat clearing, tickle in the throat, and SMIT scores were significantly different between basidiomycetous fungi (+) and basidiomycetous fungi (-) patients treated with amphotericin B. Throat clearing and SMIT scores were significantly different between basidiomycetous fungi (+) and basidiomycetous fungi (-) patients treated with budesonide.	NR	Budesonide or amphotericin B, selected based on the results of sputum culture, may lead to the total management of chronic cough-related laryngeal sensation.

Descriptions of the Interventions and Outcomes Measured

Postoperative sore throat and airway-related outcomes: Five studies reported a reduced incidence and severity of POST and postoperative complications with the use of NCs in postoperative settings [21-25]. Additionally, two studies comparing NCs with placebo demonstrated that NCs were more effective than placebo (normal saline; Table 2) [22,25]. Dexamethasone was the most commonly studied NC for postoperative complications, followed by budesonide (Table 1; Figure 3).

Bronchiolitis and postbronchiolitic complications: Two studies demonstrated the effectiveness of NCs, specifically budesonide and beclomethasone dipropionate, in reducing bronchial wheezing [26,29]. However, four studies showed no short-term or long-term clinical benefits of using NCs in bronchiolitis (Table 2). Budesonide was the most commonly studied NC, followed by beclomethasone and dexamethasone for the management of bronchiolitis [27,28,30,31]. Notably, one study reported less favorable outcomes with beclomethasone dipropionate than with 3% hypertonic saline, including higher Respiratory Distress Assessment Instrument scores, lower early discharge rates, and longer hospital stays (Table 1; Figure 3) [55].

Tachypnea of the newborn: Elfarargy et al. [19] and Vaisbourd et al. [32] reported conflicting findings regarding the use of nebulized budesonide for the management of TTN. In the study by Elfarargy et al. [19], budesonide administration resulted in improved TTN outcomes, including significantly lower Down scores, TTN clinical scores, and shorter hospital stays than placebo. However, Vaisbourd et al. [32] were unable to detect a beneficial effect of budesonide compared with placebo (normal saline) with respect to TTN clinical scores, length of hospital stay, or the requirement for respiratory support (Table 2). The budesonide dosages differed between the two studies, with Elfarargy et al. [19] using 0.25 mg and Vaisbourd et al. [32] using 1 mg.

Croup: The 10 studies assessing the use of NCs for treating croup evaluated a range of disease severities, from mild to moderate-severe croup, with most studies focusing on moderate-to-severe presentations. Nine studies [33-35,37-42] reported improvements in croup severity scores, respiratory symptoms (e.g., stridor), clinical response, or treatment outcomes such as reduced hospitalization and treatment failure rates. The studies also concluded that NCs were a safe treatment option for this condition; however, one study reported that NCs showed efficacy comparable to that of the control (L-adrenaline) in treating croup (Table 2) [36].

Meconium aspiration syndrome: Five studies [44-48] reported the effective use of nebulized budesonide in patients with MAS, demonstrating improvements in chest X-ray clearance, respiratory distress, achievement of full feeding, and reduced oxygen dependence (Table 1, Table 2, Figure 3). Across the included studies, the incidence of treatment-related complications was generally comparable between the budesonide and control groups, and no major short-term safety concerns were reported (Table 2).

Chronic rhinosinusitis: Three studies reported favorable outcomes with nebulized budesonide for rhinosinusitis, including improvements in symptom scores, nasal polyp size/score, and endoscopic outcomes compared with placebo, intranasal corticosteroids (fluticasone nasal spray and budesonide nasal spray), or a systemic corticosteroid (methylprednisolone) (Table 2, Figure 3) [50,51,53]. However, two studies showed that the effects of NCs in treating chronic rhinosinusitis were comparable to those of the control interventions (topical nasal budesonide and budesonide administered via impregnated nasal saline irrigation) [49,52].

Other respiratory conditions: Additional respiratory conditions for which NCs have been evaluated include laryngeal sensations related to chronic cough and viral wheezing [43,54]. In patients with laryngeal sensations related to chronic cough, a single dose of budesonide was associated with improvements in throat-related symptoms and symptom scores [43]. However, Clavenna et al. [54] reported that nebulized beclomethasone did not reduce wheezing episodes compared with placebo and showed no benefit in preventing viral upper respiratory tract infections or recurrent wheezing (Table 2).

Discussion

This review mapped the available evidence on the use of NCs in several medical conditions beyond asthma and COPD, highlighting areas where potential clinical benefits have been reported. A total of 36 studies were identified from the available literature and narratively synthesized in this scoping review. Around 37% of the included studies were from low- and middle-income countries. Most studies were conducted in Asia, followed by Europe, North America, and Australia, indicating a greater concentration of evidence from Asian settings. This distribution may partly reflect regional differences in disease burden and clinical practice patterns in the management of these conditions. NCs such as fluticasone and budesonide are approved by the Drugs Controller General of India for the treatment of asthma [56].

Postoperative Sore Throat and Airway-Related Outcomes

Five studies reported reduced incidence and severity of POST and postoperative complications with the use of NCs. Surgeries requiring general anesthesia and endotracheal intubation are associated with POST, an undesirable complication that occurs in 25.1%-48.8% of cases [57], leading to patient discomfort and dissatisfaction [57,58]. NCs act by reducing pain, inflammation, fluid transudation, and laryngeal edema, all of which result from mucosal inflammation around the cuff of the tracheal tube [4,59]. Overall, POST and airway-related outcomes were among the more frequently studied indications for NCs, with dexamethasone being the most commonly evaluated agent and most studies reporting beneficial effects. Across studies, NCs were compared with saline/placebo, ketamine, lignocaine, magnesium sulfate, and other corticosteroids, with most studies reporting favorable effects on the incidence or severity of POST and related airway complications.

Bronchiolitis and Postbronchiolitic Complications

Bronchiolitis caused by respiratory syncytial virus (RSV) infection is commonly encountered in infants (especially those born prematurely) and young children. The prevalence of this condition is 50%-80%, with a mortality rate ranging from 0.5% to 7% [60,61]. The acute airway inflammation seen in RSV infections in infants is similar to that observed in children experiencing acute exacerbations of asthma. This similarity provides a biological rationale for investigating the use of NCs in RSV-induced bronchiolitis and other postviral bronchitis [28]. In the present review, evidence regarding the use of NCs in bronchiolitis was variable, with some studies reporting reductions in wheezing, whereas others demonstrated no significant short- or long-term clinical benefits; one study reported no favorable outcomes with NCs compared with hypertonic saline. These findings should be interpreted in the context of contemporary bronchiolitis management guidelines, which generally do not recommend the routine use of corticosteroids because of insufficient evidence of clinically meaningful benefit [62].

Tachypnea of the Newborn

Soon after birth, some newborns present with a condition known as TTN, which is caused by delayed clearance of fetal lung fluid [63]. This condition affects 13 newborns per 1000 live births in low- and middle-income countries and 4-5.7 newborns per 1000 live births in high-income countries [64]. In most cases, the condition is self-limiting when mild, and newborns recover from TTN within 72 hours after birth. However, when severe, TTN can cause respiratory distress with cyanosis, severe retractions, tachypnea, and audible grunting [19,64]. The use of NCs such as budesonide in these newborns has been reported to improve pulmonary ventilation, reduce hospital stay, and improve blood oxygen levels, thereby reducing the need for continuous positive airway pressure treatment [19]. However, Vaisbourd et al. [32] reported that the administration of NCs did not improve the clinical course of TTN. The authors speculated that corticosteroids may act on the underlying pathogenetic mechanisms of TTN rather than its immediate clinical outcomes, suggesting a potential role in prevention rather than treatment [32]. However, evidence regarding the use of NCs in TTN remains limited and inconsistent, as only two studies evaluating budesonide were identified, with differing dosing regimens and conflicting findings, highlighting the need for further research.

Croup

Croup is a self-limiting disease commonly observed in children younger than five years, most often caused by viral infections (parainfluenza is the most common virus) but also caused by atypical organisms such as Mycoplasma. The condition affects the bronchi, trachea, and larynx and can cause laryngotracheobronchopneumonitis, laryngotracheitis, and laryngotracheobronchitis. Corticosteroids are the mainstay of croup treatment [65]. Management of croup may also include systemic corticosteroids and nebulized epinephrine depending on disease severity; however, the present review focused specifically on evidence relating to NCs. Nebulized budesonide may have a role in preventing hospital admissions and alleviating the symptoms of mild to severe croup in children [16].

Meconium Aspiration Syndrome

MAS occurs when neonates aspirate meconium-stained amniotic fluid into their lungs, leading to chemical pneumonitis, pulmonary inflammation, and surfactant inactivation, all of which obstruct the newborns' airways and result in overexpanded lungs. Nebulized budesonide was associated with improved outcomes, including shorter hospital stays, reduced oxygen requirements, and shorter durations of respiratory distress in newborns with MAS [18]. MAS was among the more frequently studied neonatal conditions, with all included studies evaluating budesonide and reporting favorable outcomes.

Chronic Rhinosinusitis

Chronic rhinosinusitis with or without nasal polyps is an inflammatory condition of the nose and paranasal sinuses characterized by persistent symptoms for at least 12 weeks [66]. NCs have been studied as a treatment option for chronic rhinosinusitis because nebulization may facilitate drug deposition in deeper regions of the nasal cavity, where the medication is retained longer and covers a larger mucosal surface area [67]. Although the Chinese Society of Allergy recommends 1 mg of budesonide daily for two weeks for the treatment of chronic rhinosinusitis [67], major international guidelines do not provide specific recommendations regarding NC delivery. The European Position Paper on Rhinosinusitis and Nasal Polyps (EPOS) 2020 recognizes nasal corticosteroids, including sprays, drops, and rinses, as the cornerstone of first-line pharmacological management for chronic rhinosinusitis [68]. However, EPOS 2020 concludes that the available evidence is insufficient to support any specific corticosteroid delivery method beyond standard nasal corticosteroids [68]. In the present review, the evidence was largely centered on budesonide, with most studies reporting favorable outcomes or outcomes comparable to those of the controls. However, a previous systematic review reported that NCs were not superior to corticosteroid nasal sprays with respect to symptom improvement, endoscopic findings, and histological outcomes, suggesting that nebulization may not offer additional clinical benefit over standard intranasal corticosteroid delivery. The same review also concluded that nebulized antibiotic delivery did not provide added value in chronic rhinosinusitis [69]. Therefore, although NCs may represent an alternative treatment approach for chronic rhinosinusitis, the mainstay of treatment remains steroid nasal sprays, and the comparative advantages of nebulized delivery require further investigation.

Other Respiratory Conditions

Nebulized beclomethasone was found to be ineffective in treating viral wheezing [54]. However, this finding is based on a single study, limiting its interpretation across different wheezing phenotypes. Regarding the management of chronic cough, because the effectiveness of NCs is based on data from a single study [43], a definitive conclusion is premature. However, the observed benefits may be attributable to the anti-inflammatory effects of corticosteroids and their ability to reduce airway hyperresponsiveness [43]. Further research involving diverse populations is necessary to establish the effectiveness and safety of NCs in managing laryngeal sensations related to chronic cough. Overall, the evidence for conditions such as laryngeal sensations related to chronic cough and viral wheezing was limited to single studies, highlighting important gaps in the current literature.

Strengths and Limitations of This Review

This scoping review is one of the first to explore and consolidate the available literature on the use of NCs in conditions other than asthma and COPD. The off-label indications were primarily evaluated through interventional clinical studies, providing clinically evaluated evidence across a diverse range of conditions beyond asthma and COPD. Many of these trials used randomized designs and incorporated blinding procedures. However, in studies involving the management of MAS, which affects neonates and infants, blinding of parents and primary caregivers was not feasible, whereas the statisticians performing the analyses remained blinded to group allocation and the interventions administered [44]. The main limitation of this review is the limited evidence available regarding the effectiveness of NCs for treating conditions such as TTN, chronic cough, and viral wheezing. The lack of substantial research on these conditions makes it difficult to draw definitive conclusions about the efficacy of NCs. The included studies demonstrated substantial heterogeneity in the reporting of procedural and patient-level clinical variables, limiting detailed contextual synthesis. In addition, heterogeneity was observed in the corticosteroid agents, dosing regimens, routes of administration, treatment durations, and comparator interventions evaluated across studies. Although several clinically relevant outcomes, including hospitalization-related measures, severity scores, oxygen requirements, and adverse events, were extracted, these outcomes were inconsistently reported across studies, limiting uniform synthesis across indications. Several included studies also enrolled relatively small numbers of participants, which may limit the precision and generalizability of the reported findings. Furthermore, the literature search was limited to the PubMed and Cochrane databases and did not include Embase, Scopus, Web of Science, or CINAHL. Consequently, some relevant studies may not have been captured, potentially introducing publication bias and limiting comprehensiveness. In addition, only studies published in English were included, which may have resulted in the omission of relevant evidence published in other languages. Consistent with the objectives of a scoping review, a formal assessment of study quality or risk of bias was not undertaken. Therefore, the findings should be interpreted as a mapping of the available evidence rather than an evaluation of its certainty or quality.

## Conclusions

Beyond their traditional use in asthma and COPD management, where they have demonstrated effectiveness, NCs show potential therapeutic benefits in selected conditions such as bronchiolitis, respiratory complications after endotracheal intubation, croup, MAS, chronic rhinosinusitis, and viral wheezing. However, the quality of the evidence remains heterogeneous and limited for several indications. Furthermore, safety findings were reported inconsistently across studies. Although several studies reported no significant safety concerns and generally favorable tolerability, others documented adverse events, highlighting the need for consistent safety reporting across indications. This review identifies important evidence gaps and areas requiring further research to better define the role of NCs across these conditions. Further research and clinical trials are needed to clarify the effectiveness and safety of NCs, support evidence-based decision-making, and improve patient-related outcomes.

## References

[1] Hodgens A, Sharman T (2024). Corticosteroids.

[2] Williams DM (2018). Clinical pharmacology of corticosteroids. Respir Care.

[3] Yang X, Jin H (2022). Safety of corticosteroids in the treatment of acute respiratory disease in children: a systematic review and meta-analysis. Transl Pediatr.

[4] Yu J, Ren L, Min S, Yang Y, Lv F (2020). Nebulized pharmacological agents for preventing postoperative sore throat: a systematic review and network meta-analysis. PLoS One.

[5] Zainudin B (1997). Steroid therapy in obstructive airway diseases. Respirology.

[6] Matalon S, Bartoszewski R, Collawn JF (2015). Role of epithelial sodium channels in the regulation of lung fluid homeostasis. Am J Physiol Lung Cell Mol Physiol.

[7] Liang TZ, Chao JH (2023). Inhaled Corticosteroids.

[8] Murphy KR, Hong JG, Wandalsen G, Larenas-Linnemann D, El Beleidy A, Zaytseva OV, Pedersen SE (2020). Nebulized inhaled corticosteroids in asthma treatment in children 5 years or younger: a systematic review and global expert analysis. J Allergy Clin Immunol Pract.

[9] Talwar D, Ramanathan R, Lopez M, Hegde R, Gogtay J, Goregaonkar G (2021). The emerging role of nebulization for maintenance treatment of chronic obstructive pulmonary disease at home. Lung India.

[10] Hagmeyer L, van Koningsbruggen-Rietschel S, Matthes S, Rietschel E, Randerath W (2023). From the infant to the geriatric patient-Strategies for inhalation therapy in asthma and chronic obstructive pulmonary disease. Clin Respir J.

[11] Fink JB, Stapleton KW (2024). Nebulizers. J Aerosol Med Pulm Drug Deliv.

[12] Melani AS (2012). Nebulized corticosteroids in asthma and COPD. An Italian appraisal. Respir Care.

[13] Maltais F, Ostinelli J, Bourbeau J (2002). Comparison of nebulized budesonide and oral prednisolone with placebo in the treatment of acute exacerbations of chronic obstructive pulmonary disease: a randomized controlled trial. Am J Respir Crit Care Med.

[14] Pleasants RA, Wang T, Xu X, Beiko T, Bei H, Zhai S, Drummond MB (2018). Nebulized corticosteroids in the treatment of COPD exacerbations: systematic review, meta-analysis, and clinical perspective. Respir Care.

[15] Zaffar MU, Ahmad W, Iqbal N (2023). Comparison of nebulized vs systemic corticosteroids for management of children presenting with acute exacerbation of asthma. Med J South Punjab.

[16] Griffin S, Ellis S, Fitzgerald-Barron A (2000). Nebulised steroid in the treatment of croup: a systematic review of randomised controlled trials. Br J Gen Pract.

[17] Marcus P, Oppenheimer EA, Patel PA (2006). Use of nebulized inhaled corticosteroids among older adult patients: an assessment of outcomes. Ann Allergy Asthma Immunol.

[18] Phattraprayoon N, Ungtrakul T, Tangamornsuksan W (2021). The effects of different types of steroids on clinical outcomes in neonates with meconium aspiration syndrome: a systematic review, meta-analysis and GRADE assessment. Medicina (Kaunas).

[19] Elfarargy MS, Abu-Risha SE, Younis RL (2020). Therapeutic effect of inhaled budesonide in transient tachypnea of newborn: a placebo-controlled study. J Popul Ther Clin Pharmacol.

[20] Tricco AC, Lillie E, Zarin W (2018). PRISMA extension for scoping reviews (PRISMA-ScR): Checklist and explanation. Ann Intern Med.

[21] Salama A, El-badawy A (2016). Does nebulized dexamethasone decrease the incidence of postextubation sore throat?: a randomized controlled study. Ain-Shams J Anaesthesiol.

[22] Mehrotra S, Kumar N, Khurana G (2017). Post operative sore throat: incidence after nebulization with ketamine, lidocaine and budesonide. Int J Med Sci Clin Invent.

[23] Kumari KS, Ashwini H, Lavanya R (2018). Comparative study of dexamethasone nebulisation with magnesium sulphate nebulisation in preventing post operative sore throat following endotracheal intubation. Indian J Clin Anaesth.

[24] Paul S, Gangwar R, Pratap KM (2019). Comparative evaluation of efficacy of nebulised budesonide with nebulised dexamethasone in reducing post-operative sore throat in patients undergoing elective lumbar spine surgery in prone position. J Clin Anes.

[25] Almustafa M, Obeidat F, Mismar A (2020). Role of preoperative dexamethasone nebulization in reducing Bougie complications encountered after sleeve gastrectomy: a prospective double-blind control interventional study. Obes Surg.

[26] Carlsen KH, Leegaard J, Larsen S, Orstavik I (1988). Nebulised beclomethasone dipropionate in recurrent obstructive episodes after acute bronchiolitis. Arch Dis Child.

[27] Richter H, Seddon P (1998). Early nebulized budesonide in the treatment of bronchiolitis and the prevention of postbronchiolitic wheezing. J Pediatr.

[28] Cade A, Brownlee KG, Conway SP (2000). Randomised placebo controlled trial of nebulised corticosteroids in acute respiratory syncytial viral bronchiolitis. Arch Dis Child.

[29] Kajosaari M, Syvänen P, Förars M, Juntunen-Backman K (2000). Inhaled corticosteroids during and after respiratory syncytial virus-bronchiolitis may decrease subsequent asthma. Pediatr Allergy Immunol.

[30] Lødrup Carlsen KC, Carlsen KH, Nikander K, Leegaard J, Havnen J, Steen-Johnsen J, Winsness A (2001). Nebulized budesonide after hospitalization for recurrent bronchial obstruction in children younger than 18 months. Pediatr Allergy Immunol.

[31] Bentur L, Shoseyov D, Feigenbaum D, Gorichovsky Y, Bibi H (2005). Dexamethasone inhalations in RSV bronchiolitis: a double-blind, placebo-controlled study. Acta Paediatr.

[32] Vaisbourd Y, Abu-Raya B, Zangen S (2017). Inhaled corticosteroids in transient tachypnea of the newborn: a randomized, placebo-controlled study. Pediatr Pulmonol.

[33] Husby S, Agertoft L, Mortensen S, Pedersen S (1993). Treatment of croup with nebulised steroid (budesonide): a double blind, placebo controlled study. Arch Dis Child.

[34] Klassen TP, Feldman ME, Watters LK, Sutcliffe T, Rowe PC (1994). Nebulized budesonide for children with mild-to-moderate croup. N Engl J Med.

[35] Geelhoed GC, Macdonald WB (1995). Oral and inhaled steroids in croup: a randomized, placebo-controlled trial. Pediatr Pulmonol.

[36] Fitzgerald D, Mellis C, Johnson M (1996). Nebulized budesonide is as effective as nebulized adrenaline in moderately severe croup. Pediatrics.

[37] Johnson DW, Schuh S, Koren G, Jaffee DM (1996). Outpatient treatment of croup with nebulized dexamethasone. Arch Pediatr Adolesc Med.

[38] Klassen TP, Watters LK, Feldman ME (1996). The efficacy of nebulized budesonide in dexamethasone-treated outpatients with croup. Pediatrics.

[39] Godden CW, Campbell MJ, Hussey M, Cogswell JJ (1997). Double blind placebo controlled trial of nebulised budesonide for croup. Arch Dis Child.

[40] Roberts GW, Master VV, Staugas RE, Raftos JV, Parsons DW, Coulthard KP, Martin AJ (1999). Repeated dose inhaled budesonide versus placebo in the treatment of croup. J Paediatr Child Health.

[41] Johnson DW, Jacobson S, Edney PC, Hadfield P, Mundy ME, Schuh S (1998). A comparison of nebulized budesonide, intramuscular dexamethasone, and placebo for moderately severe croup. N Engl J Med.

[42] Luria JW, Gonzalez-del-Rey JA, DiGiulio GA, McAneney CM, Olson JJ, Ruddy RM (2001). Effectiveness of oral or nebulized dexamethasone for children with mild croup. Arch Pediatr Adolesc Med.

[43] Ogawa H, Fujimura M, Ohkura N, Makimura K (2015). Effects of nebulized amphotericin B and budesonide inhalation for chronic cough-related laryngeal sensations. Auris Nasus Larynx.

[44] Basu S, Kumar A, Bhatia BD, Satya K, Singh TB (2007). Role of steroids on the clinical course and outcome of meconium aspiration syndrome-a randomized controlled trial. J Trop Pediatr.

[45] Tripathi S, Saili A (2007). The effect of steroids on the clinical course and outcome of neonates with meconium aspiration syndrome. J Trop Pediatr.

[46] Rangaraj S (2015). Effect of nebulized budesonide in improving the clinical outcome of neonates with meconium aspiration syndrome. J Pediatr Sci.

[47] Garg N, Choudhary M, Sharma D, Dabi D, Choudhary JS, Choudhary SK (2016). The role of early inhaled budesonide therapy in meconium aspiration in term newborns: a randomized control study. J Matern Fetal Neonatal Med.

[48] Rana KS, Konar MC, Islam K (2018). Study on effects of steroid on clinical course, short-term and long-term outcomes in neonates with meconium aspiration syndrome. J Neonatal Nurs.

[49] Thamboo A, Manji J, Szeitz A (2014). The safety and efficacy of short-term budesonide delivered via mucosal atomization device for chronic rhinosinusitis without nasal polyposis. Int Forum Allergy Rhinol.

[50] Wang C, Lou H, Wang X (2015). Effect of budesonide transnasal nebulization in patients with eosinophilic chronic rhinosinusitis with nasal polyps. J Allergy Clin Immunol.

[51] Neubauer PD, Schwam ZG, Manes RP (2016). Comparison of intranasal fluticasone spray, budesonide atomizer, and budesonide respules in patients with chronic rhinosinusitis with polyposis after endoscopic sinus surgery. Int Forum Allergy Rhinol.

[52] Dai Q, Duan C, Liu Q, Yu H (2017). Effect of nebulized budesonide on decreasing the recurrence of allergic fungal rhinosinusitis. Am J Otolaryngol.

[53] Zhang Y, Lou H, Wang Y, Li Y, Zhang L, Wang C (2019). Comparison of corticosteroids by 3 approaches to the treatment of chronic rhinosinusitis with nasal polyps. Allergy Asthma Immunol Res.

[54] Clavenna A, Sequi M, Cartabia M, Fortinguerra F, Borghi M, Bonati M (2014). Effectiveness of nebulized beclomethasone in preventing viral wheezing: an RCT. Pediatrics.

[55] Suleman M, Sadiq F, Qasim Khan M, Jabar A, Khan A, Musa N (2023). Comparison of 3% Normal Saline Nebulization versus Steroid Nebulization in the Treatment of Bronchiolitis. J Health Rehabil Res.

[56] Katiyar SK, Gaur SN, Solanki RN (2022). Indian guidelines on nebulization therapy. Indian J Tuberc.

[57] Obsa MS, Adem AO, Bancha B (2022). Global incidence and risk factors of post-operative sore throat among patients who underwent surgery: a systematic review and meta-analysis. Int J Surg Open.

[58] Fenta E, Teshome D, Melaku D (2020). Incidence and factors associated with postoperative sore throat for patients undergoing surgery under general anesthesia with endotracheal intubation at Debre Tabor General Hospital, North central Ethiopia: a cross-sectional study. Int J Surg Open.

[59] Kuriyama A, Maeda H, Sun R (2019). Aerosolized corticosteroids to prevent postoperative sore throat in adults: a systematic review and meta-analysis. Acta Anaesthesiol Scand.

[60] Greenberg D, Dagan R, Shany E, Ben-Shimol S, Givon-Lavi N (2020). Incidence of respiratory syncytial virus bronchiolitis in hospitalized infants born at 33-36 weeks of gestational age compared with those born at term: a retrospective cohort study. Clin Microbiol Infect.

[61] Justice NA, Le JK (2023). Bronchiolitis. https://www.ncbi.nlm.nih.gov/books/NBK441959/.

[62] (2026). National Institute for Health and Care Excellence (NICE): bronchiolitis in children: diagnosis and management. https://www.nice.org.uk/guidance/ng9/resources/bronchiolitis-in-children-diagnosis-and-management-pdf-51048523717.

[63] Jha K, Nassar GN, Makker K (2023). Transient Tachypnea of the Newborn. https://www.ncbi.nlm.nih.gov/books/NBK537354/.

[64] Chavan S, Malwade SD, Kumari S, Garud BP, Agarkhedkar S (2022). Incidence, clinical features, and outcomes of transient tachypnea of the newborn at a tertiary care center in western India. Cureus.

[65] Sizar O, Carr B (2023). Croup.

[66] Bachert C, Marple B, Schlosser RJ (2020). Adult chronic rhinosinusitis. Nat Rev Dis Primers.

[67] Wang C, Cheng L, Li H (2021). Chinese expert recommendation on transnasal corticosteroid nebulization for the treatment of chronic rhinosinusitis 2021. J Thorac Dis.

[68] Fokkens WJ, Lund VJ, Hopkins C (2020). Executive summary of EPOS 2020 including integrated care pathways. Rhinology.

[69] Reychler G, Domachowski C, Latiers AC, Jamar F, Rombaux P (2019). Clinical efficacy of intranasal drug delivery by nebulization in chronic rhinosinusitis: a systematic review. Rhinology.

